# Brazilian overview of nurses’ training during the COVID-19 pandemic

**DOI:** 10.1590/0034-7167-2021-0923

**Published:** 2022-07-29

**Authors:** Claudia Capellari, Joel Rolim Mancia, Edlamar Kátia Adamy, Vilanice Alves de Araújo Püschel

**Affiliations:** I Faculdades Integradas de Taquara. Taquara, Rio Grande do Sul, Brazil; II Associação Brasileira de Enfermagem. Brasília, Distrito Federal, Brazil; III Universidade do Vale do Rio dos Sinos. Porto Alegre, Rio Grande do Sul, Brazil; IV Universidade do Estado de Santa Catarina. Chapecó, Santa Catarina. Brazil; V Universidade de São Paulo. São Paulo, São Paulo, Brazil

**Keywords:** Nursing, Nursing Education, Teaching, Pandemics, Coronavirus, Enfermería, Educación en Enfermería, Enseñando, Pandemias, Coronavirus, Enfermagem, Educação em Enfermagem, Ensino, Pandemias, Coronavírus

## Abstract

**Objective:**

To present the Brazilian panorama of the training of nurses during the COVID-19 pandemic.

**Method:**

a cross-sectional study, carried out with 335 coordinators of undergraduate courses in Nursing and *online data collection,* between November 2020 and March 2021.

**Results:**

All Brazilian states were represented. Of Higher Education Institutions, 52.5% adopted remote learning within 10 days after determining social distancing and 23% after 100 days; 73.4% kept the students in a mandatory curricular internship. Practical classes had a reduction in the number of students per group (46.0%). Most faculty and students were contaminated by SARS-CoV-2 and showed worsening in mental health.

**Conclusions:**

The study identified heterogeneity in the resumption of activities, through remote teaching, which mostly occurred synchronously. There was a resumption of curricular internships and practical classes in health services, with a limitation on the number of students per field.

## RESUMO

**Objetivo:**

Apresentar o panorama brasileiro da formação de enfermeiros durante a pandemia da COVID-19.

**Método:**

estudo transversal, realizado com 335 coordenadores de curso de graduação em Enfermagem e coleta de dados *on-line,* entre novembro de 2020 e março de 2021.

**Resultados:**

Houve representação de todos os estados brasileiros. Das Instituições de Ensino Superior, 52,5% adotaram o ensino remoto em até 10 dias após a determinação do distanciamento social e 23% depois de 100 dias; 73,4% mantiveram os estudantes em estágio curricular obrigatório. As aulas práticas tiveram redução no número de estudantes por grupo (46,0%). A maioria dos docentes e discentes foi contaminada pelo SARS-CoV-2 e apresentaram piora na saúde mental.

**Conclusões:**

O estudo identificou heterogeneidade na retomada das atividades, por meio do ensino remoto, que ocorreu majoritariamente de forma síncrona. Houve retomada de estágios curriculares e aulas práticas em serviços de saúde, com limitação do número de estudantes por campo.

## RESUMEN

**Objetivo:**

Presentar el panorama brasileño de la formación de enfermeras durante la pandemia de COVID-19.

**Método:**

estudio transversal, realizado con 335 coordinadores de curso de pregrado de Enfermería y recogida de datos *on-line*, entre noviembre de 2020 y marzo de 2021.

**Resultados:**

Hubo representación de todos los estados brasileños. En las Instituciones de Enseñanza Superior, el 52,5% se incorporó a la enseñanza a distancia hasta 10 días después de la determinación del distanciamiento social y el 23% después de 100 días; el 73,4% mantuvo a los estudiantes en el estado curricular obligatorio. En las clases prácticas se redujo el número de alumnos por grupo (46,0%). La mayoría de los profesores y alumnos estaban contaminados por el SARS-CoV-2 y presentaban un empeoramiento de su salud mental.

**Conclusiones:**

El estudio identificó la heterogeneidad en la reanudación de las actividades, a través de la enseñanza a distancia, que se produjo en su mayoría de forma sincrónica. Se reanudaron las prácticas curriculares y las clases prácticas en los servicios de salud, con un número limitado de estudiantes por campo.

## INTRODUCTION

The insertion of nurses in the world of work requires the ability to lead teams, a global vision that is interconnected with technological and cultural advances^(1)^, in addition to technical-scientific skills; patient-centered care; professionalism; leadership; systems-based practice; computer and technology skills; Communication; collaborative teamwork; safety; quality improvement and evidence-based practice^(2)^.

Such indications represent challenges to be considered in the scenario of Nursing education in Brazil, taking into account the asymmetries of professional distribution and offer of courses in the country^(3)^.

With the advent of the COVID-19 pandemic and the need for social distancing, other aspects have entered the list of challenges in the training of nurses, such as the offer of curricular components, especially those that involve practical teaching in teaching laboratories and in nursing services. health; the adoption of remote learning (ER), using information and communication technologies (ICTs), which demanded support for teachers and students, demanding important adaptations from Higher Education Institutions (HEIs).

In this sense, the social distancing imposed by the pandemic has challenged the higher education community around the world. The interruption of face-to-face classes and the need to continue the activities already programmed resulted in the immediate adoption of alternative forms of teaching, especially with the use of digital tools. However, the authors defend the maintenance of the quality of nursing training in the face-to-face modality, so that ICTs are used in the curriculum in the face-to-face modality, even in an emergency situation, such as the one currently experienced due to the pandemic. Nursing is primarily a relational profession, which requires interaction, communication and interpersonal skills. The training of professionals to care for human lives demands knowledge, skills, and attitudes in the teaching-service-community integration and in interprofessional work. Therefore, nursing care is face-to-face and indispensable^(4-5)^.

The pandemic, in a way, accelerated the adoption of technology in higher education; which led to the need, in a short period, to learn its use and to develop combined learning spaces (mixed, blended, hybrid methods). In undergraduate Nursing courses, they cause some concern, as one cannot think that such technologies can serve to teach human care, nor that the student learns at anytime, anywhere, and with any technological device. It is necessary to debate nursing training in times of a pandemic, seeking strategies to strengthen the quality of training for human care^(4)^.

The return to activities remotely required teachers and students to replan the academic semester and establish pedagogical strategies supported by the use of the internet. Furthermore, considerable effort was required in order to enable strategies aimed at harm reduction in the teaching-learning process^(6)^.

In order to understand how the training of nurses was and is being developed in this pandemic scenario, at the national level, this study was guided by the following research question: how was the follow-up of activities in the undergraduate Nursing course amid the restrictions imposed by the COVID pandemic? -19?

## OBJECTIVE

To present an overview of nursing education in Brazil during the COVID-19 pandemic.

## METHODS

### Ethical aspects

Research approved by the Research Ethics Committee. Participants’ consent was obtained by signing the Free and Informed Consent Term (ICF), immediately prior to online data collection, via *Google forms*.

### Design study, time and place

This is a cross-sectional study, guided by the STROBE tool, whose data were collected from November 2020 to March 2021. The collection was carried out with coordinators of undergraduate Nursing courses in Brazil.

### Population, sample, inclusion and exclusion criteria

The study population consisted of coordinators of the undergraduate Nursing course in Brazil. For the sample calculation, the number of courses in the country was considered, understanding that, for each course, there is a coordinator. This number totals 1,246 courses, 1,236 of which are face-to-face, as recorded in the e-MEC system^(7)^. Considering a confidence level of 95%, a margin of error of 5%, and sample heterogeneity, the sample was calculated for 294 coordinators of on-site undergraduate courses from public and private HEIs.

As an inclusion criterion, it was considered to be the coordinator of a face-to-face undergraduate course in Brazil. As exclusion criteria, the following were listed: the impossibility of answering the questionnaire due to infeasibility of access to the *internet*; vacations and absences from work of any kind, and coordinator whose course has been discontinued or not started.

### Study protocol

Participants were contacted via *e-mail*, from the database of the Directorate of Nursing Education of the Associação Brasileira de Enfermagem (ABEn) National. Among other duties, this Board of Directors is responsible for presiding over the meeting of Education Directors of the State Sections and the Federal District of ABEn, which takes place on a monthly basis. The survey was disclosed in a meeting with the directors of education and the questionnaire was requested to be sent to the coordinators of undergraduate nursing courses in the respective states and DF. For courses that were not included in the records of ABEn, a search was carried out on the institutions’ websites, or telephone contact was made, with subsequent sending of an invitation by *e-mail*.

The *email* sent to the HEIs contained a brief explanation about the research; invitation to access the *link* that led to a *Google form*, containing informed consent, followed by the data collection instrument, consisting of 26 open and closed questions.

### Data analysis

Data extraction was performed directly from the spreadsheet generated by the *Google form, in the form of an* Excel® spreadsheet. After its organization, excluding duplicate responses or responses from professional education courses, statistical analysis was performed using the *Statistical Package for Social Sciences,* version 25.0.

Results are presented using descriptive statistics, in absolute and relative numbers; measures of central tendency (mean and median) and variability (standard deviation and interquartile range), with a study of the symmetry of continuous distributions, analyzed by the *Kolmogorov-Smirnov test*. The analysis of the association between categorical variables was performed using *Pearson*’s Chi-square or Fisher’s exact test. In comparing the proportions of a single variable, the chi-square test of homogeneity was used. The analysis involved the comparison of continuous variables between regions (independent groups) that were performed using the *Kruskall-Wallys - Post Hoc Dunn test.*

## RESULTS

Responses were obtained from 335 undergraduate Nursing courses in all Brazilian states and the Federal District. Table 1 presents the characterization of HEIs according to administrative category and geographic region.

**Table 1:** Characterization of Undergraduate Nursing courses according to administrative category and geographic region, Brazil, 2021

Variables	Sample (n=335) ^A^		Regions ^B^										
			North (n=31)		Northeast (n=88)		Midwest DF (n=39)		Southeast (n=89)		South (n=88)		*p*
	n	%	n	%	n	%	n	%	n	%	n	%	
Administrative category													0.034
Private	174	51.9	19	61.3	43	48.9	20	51.3	54	60.7	38	43.2	
Federal Public	74	22.1	9	29.0	21	23.9	12	30.8	12	13.5	20	22.7	
State Public	36	10.7	2	6.5	17	19.3	6	15.4	8	9.0	3	3.4	
Community	26	7.8					1	2.6	4	4.5	21	23.9	
Philanthropic	19	5.7			4	4.5			10	11.2	5	5.7	
Municipal public	6	1.8	1	3.2	3	3.4			1	1.1	1	1.1	
Grouped Administrative Category													0.006
Private	219	65.4	19	61.3	47	53.4	21	53.8	68	76.4	64	72.7	
Public	116	34.6	12	38.7	41	46.6	18	46.2	21	23.6	24	27.3	

*A: Percentages obtained based on the total sample. B: percentages obtained based on the total for each region; ¶Pearson's Chi-square test (association); §Pearson's Chi-square test (homogeneity); €Fisher Exact Test.*

For the administrative category of institutions, they were grouped into public (federal, state, and municipal public) and private (private, philanthropic, and community), a categorization that is adopted in the next analysis presented in the text.

The administrative category of institutions was compared to the five regions of the country. When analyzed in its original classification, a statistically significant difference was identified in relation to the regions (p=0.034), indicating that the North and Southeast regions were associated with private institutions; the Northeast region was related to Federal and State Public institutions; with the South region, the association took place with the Federal Public institution; the Midwest region was significantly associated with state public institutions. When the administrative category was grouped into public and private institutions, the significant association remained (p=0.006), indicating that the North, Southeast, and South regions were related to Private institutions, while the Northeast and Midwest regions were associated with public institutions.

Adherence to Remote Learning (RE) was made by 99.1% (n=332) of the HEIs. The platforms used to carry out the RE were: Google *Classroom*, 26.8% (n=89); Microsoft *Teams*, 17.8% (n=59); *Moodle*, 16.6% (n=55); Own institutional platform, 12.3% (n=41) and others, 15.8% (n=56). The type of platform used to perform the RE pointed out some items that differed significantly between regions. The *Moodle platform* prevailed in the Midwest and South regions; the Microsoft *Teams platform* was used more representatively in the Northeast and Southeast regions.

From the moment the replacement of face-to-face classes with classes mediated by digital means was decreed, while the situation imposed by the new coronavirus pandemic, the resumption of activities with the ER took place in a median time of 10 days (1st - 3rd). quartile: 3.0 - 90.0). When the number of days was approached by “time bands”, it was found that 52.5% (n=176) of the institutions started within 10 days and 23% (n=77) reported time greater than 100 days. When this data was compared to the administrative category, the median number of days was significantly higher (p<0.001) in public institutions [median: 120; (1st - 3rd quartile): 30 - 173)], when compared to private institutions [median: 7; (1st - 3rd quartile): 2 - 14)]. To resume activities with the ER, the HEIs responded about the preparation of teachers and the synchronicity of classes in this format. The details of such data are presented in Table 2:

**Table 2:** Characterization of the sample according to the number of days to start remote teaching and synchronicity of classes, Brazil, 2021

Variables	Sample (n=335) ^A^		Regions ^B^										
			North (n=31)		Northeast (n=88)		Midwest DF (n=39)		Southeast (n=89)		South (n=88)		*p*
	n	%	n	%	n	%	n	%	n	%	n	%	
Number of days until ER starts, from social distancing													<0.001¥
Mean ±standard deviation (amplitude)	51.6±75.8 (0 - 350)		60.3±75.3 (0 -250)		75.5±87.44 (0-334)		54.3±68.7 (0-196)		33.2±58.6 (0-250)		42.3±76.9 (0-350)		
Median (1st - 3rd quartile)	9.0 (3.0 - 90.0)		30.0 (3 - 90) ^A^		21.0 (7-150) ^A^		10 (4-120) ^B^		7 (4-18) ^B^		5 (2-30) ^B^		
Number of days in lanes													0.005€
Up to 10 days	176	52.5	13	41.9	31	35.2	21	53.8	53	59.6	58	65.9	
From 11 to 30 days	62	18.5	6	19.4	21	23.9	5	12.8	21	23.6	9	10.2	
From 31 to 100 days	20	6.0	5	16.1	5	5.7	1	2.6	3	3.4	6	6.8	
More than 100 days	77	23.0	7	22.6	31	35.2	12	30.8	12	13.5	15	17.0	
Preparation of teachers for the transition of the teaching modality													0.332¶
Yes	288	86.0	29	93.5	75	85.2	32	82.1	75	84.3	77	87.5	
No	47	14.0	2	6.5	13	14.8	7	17.9	14	15.7	11	12.5	
Initiative to prepare teachers for the RE													
Graduation direction or equivalent	201	60.0	22	71.0	55	62.5	19	48.7	51	57.3	54	61.4	0.033§
The nucleus of pedagogical practices or equivalent service	62	18.5	5	16.1	14	15.9	8	20.5	13	14.6	22	25.0	0.089§
Course Coordination	30	9.0	2	6.5	8	9.1	4	10.3	12	13.5	4	4.5	0.487§
There was no preparation, but general instructions	42	12.5	2	6.5	11	12.5	8	20.5	13	14.6	8	9.1	0.242§
Percentage of synchronous classes DA=1 (2.1%)													
Mean ±standard deviation (amplitude)	72.5 ± 24.1 (1 - 100)		67.9±22.5 (30-100)		69.0±229 (5-100)		63.7±26.3 (10-100)		78.4±23.1 (1-100) ^A^		75.4±24.5 (1-100)		0.001¥
Median (1st - 3rd quartile)	80.0 (50.0 - 90.0)		70 (50-80) ^AB^		70 (50-86) ^AB^		70 (40-80) ^B^		80 (70-100) ^A^		80 (60-100) ^A^		
Percentage of asynchronous classes DA=1 (2.1%)													
Mean ±standard deviation (amplitude)	27.5 ± 24.0 (0 - 100)		32.7±21.8 (0-70)		30.4±21.8 (0-80)		36.3±26.2 (0-90)		20.8±22.5 (0-100)		25.8±25.7 (0-100)		0.001¥
Median (1st - 3rd quartile)	20.0 (10.0 - 50.0)		30 (20-50) ^AB^		30 (14-50) ^AB^		30 (20-60) ^A^		10 (0-30) ^B^		21 (3-40) ^B^		

*A: percentages obtained based on the total sample. B: percentages obtained based on the total for each region; ¶Pearson's Chi-square test (association); §Pearson's Chi-square test (homogeneity); €Fisher's Exact Test; ¥Kruskal Wallys Test - Post Hoc Dunn (where means followed by equal letters do not differ by a significance of 5%). DA: number of non-responses. ER: remote learning.*

Table 3 presents the types of assistance offered to students during the ER.

**Table 3:** Assistance provided to students by Higher Education Institutions according to geographic region and administrative category, Brazil, 2021

Variables	Sample (n=335) ^A^		Regions ^B^														
			North (n=31)		Northeast (n=88)			Midwest DF (n=39)			Southeast (n=89)			South (n=88)			*p*§
	n	%	n	%	n		%	n	%		n	%		n	%		
Student Aid																	
Possibility of asynchronous access to class content	279	83.3	24	77.4	74		84.1	32	82.1		77	86.5		72	81.8		0.148§
Access to digital platforms	272	81.2	28	90.3	62		70.5	31	79.5		77	86.5		74	84.1		0.154§
Digital library	257	76.7	25	80.6	60		68.2	28	71.8		70	78.7		74	84.1		0.128§
Internet	226	67.5	25	80.6	57		64.8	24	61.5		56	62.9		64	72.7		0.105§
Training in the use of digital platforms	194	57.9	19	61.3	52		59.1	21	53.8		50	56.2		52	59.1		0.361§
Physical library	80	23.9	10	32.3	13		14.8	5	12.8		24	27.0		28	31.8		0.017§
University restaurant	10	3.0			4		4.5				4	4.5		2	2.3		- - -
			**Administrative Category**														
	**Sample (n=335) ^A^**		**Public (n=116)**							**Private (n=219)**							***p*** §
	**n**	**%**	**n**			**%**				**n**			**%**				
Student Aid																	
Possibility of asynchronous access to class content	279	83.3	99			85.3				180			82.2			0.893	
Access to digital platforms	272	81.2	87			75.0				185			84.5			0.274	
Digital library	257	76.7	74			63.8				183			83.6			0.009	
Internet	226	67.5	101			87.1				125			57.1			<0.001	
Training in the use of digital platforms	194	57.9	53			45.7				141			64.4			0.011	
Physical library	80	23.9	14			12.1				66			30.1			0.006	
University restaurant	10	3.0	4			3.4				6			2.7			- - -	

*A: percentages obtained based on the total sample. B: percentages obtained based on the total for each region. §Pearson's Chi-square test (homogeneity).*

The HEIs adopted measures to resume curricular internships after determining to replace face-to-face classes with digital means. Table 4 shows the number of days elapsed between such determination and the resumption of curricular internships, the provision of Personal Protective Equipment (PPE) for teachers and students, and the conditions for the occurrence of mandatory curricular internships.

**Table 4:** Time to return to activities and conditions related to mandatory Curricular Internships, Brazil, 2021

Variables	Sample (n=335)	
Number of days between social isolation and resumption of activities A DA=29(8.7%)		
Mean ±standard deviation (amplitude)	103.41±91.7 (0 - 400)	
Median (1st - 3rd quartile)	90.0 (0.0 - 161.0)	
Number of days between social isolation and resumption of activities in time bands A DA=29(8.7%)		
Up to 30 days	92	30.1
From 31 to 90 days	80	26.1
more than 90 days	134	43.8
Supply of PPE ^B^		
The HEIs provided the necessary PPE to the students	216	64.5
The HEIs provided the necessary PPE to the teachers	217	64.8
The Internship Granting Unit (health service) provided the necessary PPE to the students	98	29.3
The Internship Granting Unit (health service) provided the necessary PPE to the teachers	57	17.0
Students provided the necessary PPE	73	21.8
The teachers provided the necessary PPE	41	12.2
Conditions for the occurrence of mandatory internships		
The guiding professors performed direct supervision	180	53.7
The guiding professors performed indirect supervision	48	14.3
The HEIs hired nurses to carry out direct supervision	79	23.6
There was no supervision, as there were no curricular internships	45	13.4
The Internship Granting Unit allowed the return of activities, without limits of students per field	11	3.3
The Internship Granting Unit allowed the return of activities, limiting the number of students per field	228	68.1
The internship granting unit did not allow the return of activities	46	15.0

*DA: number of non-responses. A: percentages obtained based on the total sample. B: Percentages obtained based on the analysis of the number of occurrences of cases (multiple response question). HEIs: Higher Education Institutions; PPE: Personal Protective Equipment.*

Table 5 shows the number of students in compulsory Curricular Internship and included in the “Brasil Conta Comigo” Strategic Action, in the first and second semesters of 2020 (AEBCC) ^(9)^.

**Table 5:** Number of students in compulsory Curricular Internship and in the Strategic Action Brazil Conta Comigo, Brazil, 2021

Variables	Sample (n=335) ^A^	
Compulsory Curricular Internship		
Number of students in Curricular Internship in 2020/1	246	73.4
Mean ±standard deviation (amplitude)	62.9±127.4 (1 - 1450)	
Median (1st - 3rd quartile)	32.0 (21.0 - 60.0)	
Number of students in Curricular Internship in 2020/2	256	76.4
Mean ±standard deviation (amplitude)	60.7 ±97.6 (2 - 970)	
Median (1st - 3rd quartile)	34.0 (21.0 - 63.0)	
Strategic Action Brasil Conta Comigo		
Number of students at AEBCC in 2020/1	105	31.3
Mean ±standard deviation (amplitude)	28.8±80.4 (1 - 700)	
Median (1st - 3rd quartile)	10.0 (3.0 - 22.0)	
Number of students at AEBCC in 2020/2	91	27.2
Mean ±standard deviation (amplitude)	31.7 ±84.8 (1 - 700)	
Median (1st - 3rd quartile)	12.0 (4.0 - 30.0)	

*A: Percentages obtained based on the total of the sample.*

The participants, when asked about how the practical classes held in teaching laboratories and health services took place during the period of the new coronavirus pandemic, informed the data presented in Table 6.

**Table 6:** Realization and conditions of practical classes in the laboratory and in health services, Brazil, 2021

Variables	Sample (n=335) ^A^	
Laboratory practices and skills training ^B^	**n**	**%**
Laboratory classes were replaced by theoretical classes, via digital platforms	119	35.5
Laboratory classes were not held	115	34.3
The laboratory classes were carried out remotely: the student at home and the teacher in the laboratory, synchronously	104	31.0
The laboratory classes were carried out remotely: the student at home and the teacher in the laboratory, asynchronously	43	12.8
Others	133	39.7
Practical classes in health services ^B^		
The practical classes continued with their usual occurrence	16	4.8
Practical classes had a reduction in the number of students per group	154	46.0
The HEIs provided the necessary PPE to the students	139	41.5
The HEIs provided the necessary PPE to the teachers	150	44.8
Health services provided the necessary PPE to students	30	9.0
Health services provided the necessary PPE to teachers	17	5.1
Students provided the necessary PPE	43	12.8
The teachers provided the necessary PPE	17	5.1
The professors performed direct supervision	114	34.0
The HEIs hired nurses to carry out direct supervision	31	9.3
Health services allowed the return of activities, with no limits on student per field	5	1.5
Health services allowed the return of activities, limiting the number of students per field	135	40.3
Health services did not allow the return of activities	58	17.3
There were no practical classes in health services	145	43.3

*A: Percentages obtained based on the total sample; B: Percentages obtained based on the analysis of the number of occurrences of cases (multiple response question). HEIs: Higher Education Institutions; PPE: Personal Protective Equipment.*

The participants’ description of the other alternatives adopted for laboratory practice and skills training included: the removal of *kits* to carry out skills training at home; the recording of videos, by the students, for teacher evaluation; the realization of practical classes in the laboratory, with adequacy in the number of students, in order to respect the biosafety protocols; the extension of practical classes; the reduction of the practical workload; the use of simulation and/or virtual laboratories; classes in the hybrid model; adoption of case studies, lectures, dramatization, *debriefing* of film scenes and recording of the procedures by the professors for availability to the students.

Participants were also asked if there was the preparation of the faculty and students, in relation to the necessary care to face the COVID-19 pandemic, before returning to the practice fields and the vast majority responded positively (80.6%; n =262). When asked to briefly describe how this preparation took place, the use of videos and explanatory texts on biosafety, remote and face-to-face training, monitoring of the use of PPE, PCR testing for SARS-CoV-2, inclusion of guidelines in an institutional manual, training courses, creation of standard operating protocol and flows, meetings, as well as guidance and support in mental health.

As for aspects related to contamination by SARS-CoV-2 and worsening of mental health as a result of the context experienced by the COVID-19 pandemic, the results showed that 89.3% of students and 77.9% of teachers were contaminated by SARSCoV2, as well as 82.1% of students and 70.5% of professors showed worsening of mental health.

As for the support offered by the HEIs to the faculty and students who showed a worsening in their mental health status, the following were cited: “hosting strategies” (n=200, 56.3%), “individual psychological care” (n=189, 53 .2%), “educational material” (n=125, 35.2%), “referral to a reference health service” (n=91, 25.6%), “support group” (n=84, 23.7%) and “collective psychological care” (n=50, 14.1%).

## DISCUSSION

Regarding the nature of the training institution, the findings of this study are consistent with the characteristics pointed out by the Profile of Nursing in Brazil survey, which shows that 57,4% of Brazilian nurses were trained by private HEIs and 35,6% by public institutions. The same publication shows a significant increase in the participation of private HEIs in Nursing training, especially after the 1990s^(3)^. The privatization of nursing training is recent and progressive^(1)^, accounting for 75,7% of nurses who graduated between 2010 and 2013^(3)^. Regardless of the nature of the training institution, unlike Brazil, many countries do not even have regulatory mechanisms for Nursing education^(10)^.

With regard to the geographical location of undergraduate nursing courses, the data were partially similar to the region where Brazilian nurses graduated, who are trained in the North (5,8%), Midwest (7%), South (13,2%), Northeast (24,2%), Southeast (49,7%), consolidating the Southeast region as hegemonic, by concentrating the training of half of the country’s professionals^(3)^. At the same time, it is necessary to consider the inequality in the distribution of professionals in relation to the place of training and professional performance. In Brazil, the density of nurses was 24,54 professionals per 10,000 inhabitants in 2018; however, when this analysis is performed by federation unit, an important discrepancy is observed, such as, for example, the state of Maranhão, which has a density of 14,13, and São Paulo, with 43,39^(11)^, which directly impacts the attention to the needs of the population.

With the COVID-19 pandemic installed, the need for nurses has intensified around the world, as well as the indispensability of training new professionals. In this context, adherence to the ER was practically inevitable for Nursing education, which was also necessary for other courses in the health area, such as Medicine, and it was configured as the only viable pedagogical strategy during the COVID-19 pandemic^(12)^. Therefore, in addition to teacher preparation, teachers needed to be involved in the replanning of activities, in the appropriate feedback and evaluation, adapted to the online model, in addition to the flexibility of educational models, which traditionally took place in person and based on interpersonal relationships^(12)^.

The RE adopted by the HEI during the pandemic presented itself differently, with a return in less than 10 days in more than 50% of the HEIs. This return can be configured as Emergency Remote Teaching (ERE), used in crisis situations, catastrophes, and is configured as a temporary and sudden change. It is remote because students and teachers cannot go to the classroom, and emergency, because there was no planning time and no one was prepared for this type of teaching. In the ERE, the same professors, the same students, and the same number of students continue, and the professor is the one who creates and organizes his classes, intermediating via technology^(4)^.

When teaching strategies focus on learning and advance in terms of quality, ERE moves towards intentional remote teaching (ERI). In the ERI, the professors, the pedagogical team, and the managers think, plan and elaborate this type of teaching. There is an organizational process of this teaching^(4)^.

The results show that 47.5% of the HEIs waited a period longer than 10 days for this return, leading to the understanding that these HEIs proposed the ERI, which may denote learning intentionality and not simply the delivery of content or delivery of classes. In this sense, it is worth reflecting on the proportionality of synchronous and asynchronous classes. The synchronous classes take place with the intermediation of the teacher with the student in real-time, using ICTs, while the asynchronous classes correspond to the contents that are stored on digital platforms, which allow the student to access the content at the moment they define. There is no synchronization between teacher and student in real-time. However, the professor uses communication strategies with the student to solve their doubts and indicate the materials to be consulted/studied. It can be inferred, therefore, that the results presented here lead to asynchronous remote teaching, for the most part, reflecting a concern regarding the quality of Nursing training, different from those identified in the meta-synthesis of previous pandemic situations, in which, of the seven studies included, four used recordings to conduct online classes^(13)^.

There has been progress in the technology market, with an increase in the number of available platforms becoming evident. These platforms have different strategies for teaching, both as a repository of materials and content, as didactic tools used in the educational process, as well as those that enable students’ assessment processes. It is important to highlight that the need to include ICT in undergraduate nursing education has been pointed out for many years, however, the pandemic accelerated this process, which made the HEIs also accelerate the training processes of teachers for the use of these platforms and technologies. Other publications present examples of used platforms, such as Microsoft Teams and Canvas^(14)^ and Google Classroom^(15)^. It should be noted that, in addition to these, there are platforms for videoconferencing, which were not investigated in this study.

Like Nursing, Medicine also needed to implement pedagogical strategies for the continuity of curricular medical education, which was based on remote teaching and ICT^(12)^. The authors draw attention to the fact that, although the use of such technologies is inevitable, the speed with which they were implemented during the COVID-19 pandemic can generate intellectual gaps in the training of students, especially because, in the texts evaluated by them, there were omissions about limitations and weaknesses of the strategies implemented, which may be related both to the recent nature of their adoption and to the disregard of inequities in access and underdeveloped realities. On the other hand, the advance in the use of digital technologies is undeniable, which has seen innovation and new possibilities in virtual education^(16)^.

In the pandemic context, it is worth noting that a significant number of HEIs provided assistance to students, which ranged from training in the use of digital platforms to internet access. In a study carried out with Indian dentistry students, it was identified that 93% of them considered that, in the context of the pandemic, online teaching is the best way to learn^(17)^. On the other hand, a survey carried out with medical students from Brazil, the Republic of Cape Verde, and Guinea-Bissau, also during the COVID-19 pandemic, identified that 8,65% of the participants stated that both the equipment and the available internet did not would allow monitoring the activities in RE, and 19,2% judged the realization of RE unfeasible. Among the participants, there was a statistically significant difference between the speed of the internet and compatible equipment for performing the RE, although, in the context of medical students, low-income students were the minority^(18)^.

In a study carried out with nursing students and professors, 57,8% expressed a preference for face-to-face teaching. Some participants cited difficulties related to the lack of training in the use of ICT, access to the Internet, and availability of equipment^(19)^. Thus, it is considered that the discrepancy of access to digital technologies makes the availability of equipment and means of access to the internet to students appropriate and necessary.

For students in the health area, however, the RE is not enough for training that allows adequate health care for people and population groups, especially in times when the training of nurses urges recalibration in education and in the planning of forces. worldwide^(10,20)^. Thus, it was up to schools and courses to implement measures that would allow for mandatory curricular internships, which are essential in health services, as well as practical classes in laboratories and health services. To this end, there was a need for greater caution, preparation of health services and the academic community, as well as the provision of materials that would allow students and professors to transit through the scenarios in question. Thus, the difference between the time of resumption of theoretical classes in the ER model and the resumption of curricular internships, which took a median of 90 days to be restarted, is remarkable, with most HEIs, even if in a heterogeneous way, offering preparation of teachers and students about the necessary care in relation to COVID-19.

Both for the resumption of curricular internships and practical classes in health services, part of the HEIs provided PPE to teachers and students, an essential condition for the occurrence of such activities, not only due to the unsanitary conditions, but due to the COVID-19 pandemic. Although for curricular internships, the granting party is responsible for the provision of PPE^(21)^, it is possible that there was difficulty in this offer, given the scarcity^(22-23)^ and abusive increase in prices of such materials in the pandemic scenario. It is worth noting that, although it was possible to return to practical activities in health services, the possible need to provide their own PPE, travel to places, and other expenses with academic training may have caused difficulties for students, given the social disparities possibly present.

In an unusual and challenging scenario, it was found that a significant part of the health services allowed the return of internships and practical Nursing classes, limiting the number of students per field. It should be noted, however, that 4323% of the HEIs did not resume practical activities in health services in the period, which may be related to the care not to overload the services, not to cause agglomerations and not to expose students to risks, taking into account that the practical classes are carried out predominantly in small groups. For those who returned, there was a lower percentage of supply of PPE to professors and students by the HEIs, in relation to curricular internships.

It is interesting to see how the practical laboratory classes were reorganized, for which it was mentioned since their non-occurrence, replacement by theoretical classes, and the occurrence in a synchronous way, via digital platforms, with the teacher in the laboratory and the student at his home. At this point, it is highlighted how quickly digital technologies were adopted and served as a support for the continuity of education, enabling teaching in the health area, such as the report of teaching anamnesis, assisted by digital technologies^(24)^, and a training program based on clinical simulation for Brazilian hospitals or academic institutions, at the time of the pandemic^(25)^.

Regarding the mental health of students, a recent study reveals that emotional reactions described by stress, anxiety, grief, anger, and panic were observed, associated with concern about delaying academic activities and fear of getting sick. The pandemic and aspects related to it interfere with the academic life and health of students and their families, causing negative effects on mental health^(26)^, which corroborates the findings of the present research, which indicated a worsening in the mental health of students and teachers. Characteristics to be considered in this context also concern sociodemographic differences, since Brazil is a country of continental dimensions and heterogeneous. In the United States, research carried out with 355 Nursing students identified a statistically significant relationship between stress and stressors related to online teaching during the COVID-19 pandemic and the sociodemographic characteristics of students^(27)^.

Just as students reveal the negative effects on their mental health, teachers also point out psychological suffering because of the pandemic. A study points out that the main factors responsible for such effects are related to little or no ability to use ICT in the teaching-learning process and the lack of mastery of technology, associated with the context of self-demand and pressure by HEIs and the need to insert it in pedagogical praxis. Furthermore, work overload related to domestic obligations results in a greater risk for the development of psycho-emotional suffering, which can worsen and progress to mental illness^(28)^ . Although it is possible to use strategies to help people affected by this condition, in the context of the pandemic, such as those identified in this study, such interventions still have a low level of evidence^(29)^.

Although Brazil is configured as a country with great territorial extension and diversity, it can be said that there was similarity in terms of the panorama presented by the HEIs, in the general context, when compared in their administrative category.

### Study limitations

The limits of the study refer to regional differences, as it was not possible to control the proportionality of sample responses by geographic region. In addition, having investigated the coordinator’s point of view, teachers from different areas of activity (theoretical and practical), as well as students, were not included, which indicates the need for research that includes such actors.

### Contributions to the field of Nursing

This study contributes to the knowledge of the panorama of professional training in Brazil in times of the COVID-19 pandemic and it is possible, through communities of practice, to share successful experiences to overcome the obstacles faced, as well as the adversities that permeate the time course.

## CONCLUSIONS

The study presented an overview of nursing education in Brazil during the COVID-19 pandemic, from the perspective of 355 course coordinators from all Brazilian states and the Federal District. Most participants were from private HEIs, followed by federal public ones.

With social isolation due to the pandemic, the resumption of teaching activities through the ER occurred heterogeneously between public and private HEIs. In most HEIs, teachers were prepared for the transition from face-to-face to remote teaching, which started to occur mostly synchronously, via digital platforms. However, synchronicity occurred heterogeneously in different regions of the country.

Among the types of assistance provided to students by the HEIs, those related to digital technologies stood out, with the digital library, internet and training for the use of digital platforms statistically differing between public and private, but not differing between regions of the country.

For the resumption of practical activities, most HEIs offered preparation of the academic community. Curricular internships were restarted at a median of 90 days after social distancing was enacted. To this end, PPE was provided by the HEIs or internship granting units to professors and students. A percentage of students and teachers needed to provide their own PPE. Supervision of interns was carried out directly or indirectly. Most of the internship granting units allowed the return of activities, limiting the number of students per field.

Regarding the practical activities in the laboratory, the alternatives pointed out by the coordinators were the replacement by theoretical classes, their non-performance, the occurrence in a synchronous way, via digital platforms, with the teacher in the laboratory and the student at home. As for the practical classes in health services, 46% of the participants reported a reduction in the number of students per field. For these occurrences, there was a lower percentage of supply of PPE to professors and students by HEIs, in relation to curricular internships.

A considerable part of the participants reported that there was contamination of students and teachers by SARS-CoV-2, who had worsened mental health due to the pandemic.

New studies are needed to assess the quality of teaching and the impact of initiatives used to train nurses in this context.
